# A Manual of Medical Diagnosis

**Published:** 1858-05

**Authors:** 


NORTH AMERICAN

### MEDICO-CHIRURGICAL REVIEW.

MAY, 1858.

### Analytical and Critical Reviews.

ART. I.—*A Manual of Medical Diagnosis; being an Analysis of the Signs and Symptoms of Disease.* By A. W. BARCLAY, M.D., Cantab. et Edin., Fellow of the Royal College of Physicians, Assistant Physician to St. George's Hospital, etc. etc. Philadelphia: Blanchard & Lea, 1858, pp. 423, 8vo.

THE medical literature of England and America is poor in works on Diagnosis. In this respect it suffers by a comparison with that of France, which, on the contrary, abounds in works of this kind. The first systematic treatise on the subject, to which the medical public of this country had access, was in the shape of a translation from the French of Martinet's "*Manuel de Clinique Médicale.*"* The next, and the only original English production, before the appearance of the one which is soon to claim a notice at our hands, is that by Marshall Hall, entitled "The Principles of Diagnosis," two editions of which were brought out on this side of the Atlantic; the second appearing in 1835. The third treatise was a translation from the German of "Schill's Outlines of Pathological Semeiology," republished, from the English edition, in Bell's Select Medical Library, (1841.)

In France, the improvement in medical education, which followed the new organization of the schools, brought about by the Revolution of 1789,

* The title of the translation was somewhat different from this. Our copy is mislaid, and we cannot give its date, which, we think, was 1826.

was evince in a greater attention to diagnosis, in connection with, or, rather, as an integral part of medical and surgical clinics. From the date (1808) of Landré Beauvais' work to the present time, we have to record the names of Double, Martinet, Rostan, Piorry, and Raciborski, as authors of treatises on diagnosis, or on diagnosis and prognosis, of various degrees of size and merit.* The works on the diagnosis of particular classes of disease, as of the heart and respiratory organs, either separate, or conjoined with other parts of their pathology and treatment, are still more numerous since Laennec led the way in this department. In some French treatises on pathology, the subject of diagnosis occupies a considerable portion of the entire work. Thus, for example, in the *Traité Elementaire de Pathologie Interne*, by MM. Hardy and Behier, five hundred pages of the first volume are taken up with the rules of diagnosis proper and of prognosis, and their detailed application to Semeiology. Chomel devotes three-fifths of his *Pathologie Générale* to the department of diagnosis. Among American writers, Dr. A. Stillé gives deserved prominence to diagnosis in his work on Pathology.

If poverty implies want, and, at the same time, an eager desire for relief, then ought medical readers in the English language, on both sides of the Atlantic, to be grateful for the present volume by Doctor Barclay, which, in its structure and details, is of such a nature as to minister to their necessities. Were we required to indicate the greatest deficiency in the practice of medicine in these United States, we should be obliged to say hasty and imperfect diagnosis. Nor can it well be otherwise under the neglect of regular and systematic teaching of pathology, of which diagnosis forms so important a division. We scarcely need a stronger proof of the neglect of a thing than ignorance of its relations to other things; and by this rule it will be found that diagnosis is not generally known or admitted to be an integral part of pathology; and in close relation with a knowledge of the material and morbid changes in the structure of the organs, another branch of pathology, known under the title of morbid or pathological anatomy. Examination of the changes of structure affords an invaluable confirmation or correction of our diagnosis, and hence the propriety of an author cited above, Marshall Hall, declaring, in the very opening of his work on diagnosis, that one of its sources is morbid anatomy. To the study of this latter, he thinks, we are principally indebted for the recent progress, and, indeed, for all that is solid in medical science. But on this point we need not dilate, after the full expression of our views in the initial article of this journal for January last. The other and third division of pathology, viz. etiology, is often spoken of among us as a dis-

* Those by Double, Rostan, and Piorry are, each, in three volumes.

tinct branch. Demanding, as it does, an inquiry into the circumstances or the causes under which disease is produced and developed, and assumes certain characters, its relations to diagnosis become sufficiently evident.

It is generally conceded that correct diagnosis furnishes the only sure indications for the safety if not success of therapeutical treatment; and if this be so, we cannot say much for the probabilities of successful results in American practice. We can hardly be expected, in a spirit of self-condemnation, to quote approvingly the maxim of Hippocrates: "*Qui ad cognoscendum sufficit medicus, ad sanandum etiam sufficit;*" or the words of Baglivi, to the same purport: "*Qui benê judicat, benê curat.*" We may endeavor, indeed, to find consolation for our shortcomings in this matter, by saying, that a very minute diagnosis would often seem, as in the case of French and other physicians on the continent of Europe, to be followed by, if it does not absolutely lead to, inert practice; while we, even although we do make a very hurried and crude examination of the symptoms of disease, and often mistake the seat of the lesion, give remedies with a free hand, and produce marked effects. If energy of treatment be a measure of success in the contest with disease, we might claim superiority over the learned and the accurate in diagnosis; but it must be admitted, that a fortress is not driven to surrender, nor an enemy defeated in the field of battle, by the mere expenditure of a large amount of powder and projectiles fired off at random, or in the air; but, rather, by the choice of distance and correctness of aim, before the guns, great and small, are discharged. May not our heroic modes of treatment of diseases, as we sometimes boastfully term them, be compared to the knock-down style of argument, which, though very striking and very forcible, is not commonly regarded as the most logical or convincing, especially if it have been practiced on the wrong person? The patient who receives a hundred grains of calomel into his stomach in the course of a few days, for supposed hepatitis, when, in fact, he is laboring under pneumonia, will not be particularly cheered by learning that, under a mistaken diagnosis, he has been the subject of a very energetic treatment, and has exhibited considerable powers of endurance. Nor will another have cause for gratulation at having been bled to syncope for hemoptysis, and bled largely again on the recurrence of the hemorrhage, under a belief, on the part of his medical adviser, that it is a primary disease, when a careful diagnosis would have shown it to be a symptom of tuberculous formation in the upper lobes of the lungs. Gradually increasing debility and emaciation, with loss of appetite and impaired digestion, may suggest to the superficial observer a call for tonics and stimulants and a rich diet, whose views and practice would be not a little modified were he to carry out his diagnostic investigations, and make the discovery that he has to deal with a case of albuminuria. A female complains to us that she is dyspeptic and nervous, and easily fatigued after slight exercise, and perhaps she may timidly add that she has frequent calls to urinate. Shall we put her on a course of alkalies, and nervines, and tonics, and special diuretics? Such things are not unfrequently done, but with only slight and transient benefit, until more careful inquiries are made by a person who is intent on obtaining a full diagnosis, and who suggests a relaxation and partial prolapse of the uterus, which, after permission granted, he verifies. A treatment indicated by the diagnosis soon restores this woman to comfort, and after awhile to health, and ability to take a full measure of exercise. We shall not multiply examples to the same purport, but proceed to notice analytically the Manual of Doctor Barclay.

The author, in a short preface, while he acknowledges the success with which diagnosis is cultivated on the continent of Europe, cannot refrain from coupling with this praise a censure for inefficient practice by the same parties. We have just now touched on this point, and we would ask, without further argument, whether it has been settled by medical statistics. We are not aware that it has been; and, in the interim, we may be allowed to doubt the validity of the judgment, even at the risk of our being accused of want of patriotism, for the American plea is the same as the English one. In a full introduction we are told that—

"All true diagnosis is ultimately based upon inductions separately framed out of clinical and pathological investigations and experiments. By careful and repeated observation, we have succeeded, with every appearance of truth, in associating certain phenomena observed during life with particular lesions found after death; and these form the first step in our progress. Sound principles have advanced exactly in proportion to the number and the accuracy of these conclusions, because there are many conditions which we are not yet, and perhaps never shall be able, to associate with any appreciable change in structure; and to them we must apply by inference the truths which have been taught in other instances by direct observation. In so far as we are able correctly to interpret symptoms, and to trace out in connection with them a real change of structure or of function which affords an adequate explanation of their presence, in so far are we prepared to form a correct diagnosis. It is not the province of this branch of study to elucidate the *modus operandi* of the change; but, assuming these principles as true, our especial work is to learn to group symptoms together, and to analyze them separately in such a manner that we may be able to apply to them a scheme already supplied to our hand, which shall in some way account for their existence. It is by the nature of this assumption that rational medicine is distinguished from empiricism. The latter equally seeks to group symptoms together, and to assign to each group the most suitable remedies; but the theory or scheme which it furnishes is not based on scientific principles. In the application of the theory to the case under observation, the two are exactly analogous. A comparison is to be instituted between the probable results of the supposed malady and those presented by the particular case, and their correspondence serves for the verification of the hypothesis. In short, it is the deductive process of reasoning applied to the elucidation of morbid phenomena. We gather together in the best manner we can the fragmentary evidence of symptoms, and we apply to it the known laws of causation taught by the theory of disease." P. 21.

The correctness with which diagnosis is performed depends on a variety of circumstances; the amount of evidence obtained by methodical investigation, or assigning of the true value to each portion of the evidence, especially if the group of symptoms be of a very complex character, and an accurate knowledge of the theory of disease. While abbreviating the argument we use nearly the words of the author. From these considerations, he goes on to say, it must be evident that the more numerous and the more simple the symptoms presented to our notice, the more certain must be our diagnosis. Attention to one symptom only cannot lead to truth, since the causes of its production may be various; but when a greater number are considered, and are found to harmonize together, the possibility of the whole group being produced by one or other of the several causes becomes, necessarily, very greatly diminished. A first examination does not always suffice for our determining the true value of the symptoms or the precise character of the disease,—not only on account of possible oversight on our part, but, also, of the disease not having fully developed itself. Another point must also be kept in view in diagnosis. Diseased action in the body is very complex, and the phenomena present may not be all reducible to the results of one form of disease or the morbid condition of one set of organs; it may, on the contrary, be compounded of the effects of several causes acting together. Circumstances concomitant with the symptoms, which may seem to point clearly to a particular condition, we will suppose inflammation, of an organ, are not to be overlooked, such as the evidences of strength or weakness, increased or depressed vitality. Diagnosis is not meant to furnish us with abstract propositions, but to be an aid to us in restoring the patient, who is the subject of our investigation, to health.

The first chapter of the volume before us treats of the method of diagnosis, the history of the case under examination, the narrative of previous symptoms, arrangement of existing phenomena, plan of carrying on the investigation, the necessity of a classification of diseases to diagnosis, and a table of diseases. The history naturally divides itself into two parts: the report of the patient himself, or of his friends and attendants, of what happened before he was seen by the physician; and the phenomena actually observed at the time of examination. The same distinction must be made between events occurring in the absence of the observer, and those noted at any subsequent visit. The previous history is often of great importance; it ought to commence with the very first deviation from health, in so far as the sensations and functions of the patient are concerned, and it ought to give a connected account of the changes which have subsequently transpired. This account is of itself sometimes sufficient to point out the nature of the malady.

"This preliminary investigation leads to the association of symptoms according to their order of sequence, and we must be careful, by observing them from another point of view, to correct any false impression to which it may have given rise. While, therefore, we follow the patient telling his own case in his own way, it is quite essential that we should make a subsequent and independent investigation of existing symptoms according to some systematic course, which shall have the effect of ranging them in such scientific groups as may most readily and naturally lead to the detection of the cause which best accounts for their origin, and most fully satisfies all the requirements of the case."

In seeking for such an arrangement, there are two classes of indications, the general and the local; but these two are so inextricably mingled together, that no more can be done than merely to adopt, as far as possible, the general indications first and the special indications afterward. A plan which the author deems best adapted for obtaining the information required is now given. In a similar order he discusses the diseases of which the plan furnished the indications, and with this view he introduces the classification which he believes to be most serviceable, and which he follows in the remaining or chief portion of the volume.

The duration and sequence of phenomena constitute the subjects of another chapter, under the heads of division of diseases into acute and chronic; long ailment; pain, in reference to duration; order of sequence; and established course of disease.

Chapter III. is on the "General Condition of the Patient." It treats of objective and subjective phenomena; general symptoms; skin; pulse; tongue; bowels and kidneys; thirst and hunger; appearance; position or posture; sensations; particular signs.

"Objective phenomena, in their relation to a general state, are those changes in the condition of vital functions of which the observer becomes conscious by his own perceptions. * * * * As independent of the sensations or imagination of the patient, this class of phenomena is less under the control of his volition; and, therefore, less liable to be simulated or exaggerated.

"Subjective phenomena have special reference to the sensations of the patient; they express, to a certain extent, his consciousness of general derangement of vital functions; but their more direct tendency is to point out the particular function which is disturbed, and hence the particular organ or portion of the body where disease is located. The two classes are, in a great measure, inseparable. They may be divided into the four following groups:—

"1. General symptoms, as pertaining to—

*a.* Temperature and dryness of skin;

*b.* Fulness and quickness of pulse;

*c.* Appearance of the tongue;

*d.* State of bowels and kidneys;

*e.* Desire for food and drink.

"It is indispensable to a correct result, that the whole of these should be always taken together, as the indications derived from one source serve to correct those drawn from another, and any one of them is valueless as standing alone.

"2. The general appearance of the patient:—

*a.* Size, including emaciation, and increase of bulk, whether general or local;

*b.* Aspect of face, and expression;

*c.* Changes of color of skin, general and local.

"3. His position, or posture:—

*a.* In bed; the manner of lying—on the back, on either side; quiet, restless, etc.

*b.* Out of bed; posture, gait, stiffness or loss of power of limbs.

"4. The sensations of the patient."

Pertinent remarks follow on the several points included in these groups, inculcating sound lessons for careful observation and inquiry at the bedside of the patient. To the student they are full of monition; to the hurrying routine practitioner, full of rebuke. Then come particular indications derived from each group in succession. In the first we have the skin, the pulse, the tongue, the character of the stools, the character of the urine, the various conditions of the appetite, thirst. The particular indications in the second group are derived from emaciation; remarkable increase of bulk; the aspect, which is full of significance; expression; alteration of color. The more important of the particular indications from group third are, positions in bed, posture, and gait. Indirect but far from unimportant indications are furnished from the fourth group, viz.:—

"*a.* The contrast in genuine cholera between the corpse-like coldness of the body, and the sensation of heat with which the patient is oppressed; in diarrhœa there is generally chilliness.

"*b.* As a sensation of an opposite kind, may be mentioned the common complaint of chilliness in fever, when the skin is burning hot.

"*c.* The sensations of the hypochondriac are opposed alike to the evidence of the senses and the conclusions of reason.

"*d.* A patient's complaint of want of sleep is almost certain to be exaggerated: the report of the nurse or attendant can alone be relied on.

"*e.* The sympathetic pains form an important group. Thus, pain of the right shoulder may proceed from disease in the liver; pain of the sacrum, from disease of the uterus; of the thigh and testicle, from nephritis or nephralgia; of the knee, from disease of the hip; of the meatus, from stone in the bladder, etc.

"*f.* Complaints of pain are often exaggerated in persons of nervous susceptibility."

It will have been seen, that some time and much attention ought to be given by the physician to an observation of the general condition of his patient, as elucidated by certain symptoms and signs, before he proceeds to a special investigation of the direct nature and causes of the disease. For this purpose, a rapid survey is to be made of the different organs, while examining more closely any one in which evidence is given of an abnormal state, by the sensations of the patient or by facts elicited during an inquiry into the history of the case. But we find in a large class of diseases that the abnormal sensations and disturbance referred to in one or more organs are not always to be received as evidences of positive lesion or alarming functional derangement; but rather of that general disturbance of the entire economy which are known under the designation of fever. The diagnosis of "Febrile Diseases" is opened to us in Chapter IV. under three divisions:—In the first of these the author speaks of a general febrile state—fevers, continued fevers, epidemics, cutaneous spots, subdivisions, complications, remittent fever, influenza, epidemic cholera and its relation to diarrhœa. Division second gives eruptive fevers, measles, scarlatina, varioloid, erysipelas; and division third intermittent fevers.

It is mentioned, as a matter of surprise, "how great a similarity all cases of fever have to each other for a given time and in a given place; and how much they differ from cases occurring at another time or in another locality." Among the modes of division of fevers "is that obtained from the presence and absence of cutaneous eruption and its special characters." Having described the three varieties of the true fever-spots, each of which may be mingled with petechiæ, the author says:—"Of this classification it is most important to note that there is no character of disease with spots, of any one of the fevers noticed, that may not have its exact counterpart in a case where there are no spots at all." Another classification is derived from the prominent symptoms which occur simultaneously; and fever is spoken of as fever with head symptoms, fever with chest symptoms, and fever with abdominal symptoms. Complications, symptomatic or simply concomitant, of fever next come up. The author seems to show that in continued (typhus fever) the prominent feature is not inflammatory, although local congestions occur in its course, and, in consequence of the irritation thus caused, a sort of inflammatory action may be induced. In the head, we have, he tells us, delirium, insomnia, unconsciousness, coma, which, as the history of the case shows, are not inflammatory. In the lungs, congestion almost always comes on more or less from position, and especially in those cases where the blood is most altered in character.

The slight acquaintance of the author with remittent fever is exhibited in his telling us:—"It is now very generally believed to be only typhus as modified by atmospheric influences and the condition of the nervous and sanguiferous systems of Europeans residing in tropical latitudes. The same analogy holds with reference to the only fever of this type ever seen among ourselves—infantile remittent." Unless we are greatly mistaken, the countrymen of Dr. Barclay, members of the profession, who are familiar, from long residence in the East and West Indies, with remittent fever, would hold very different language in regard both to its etiology and symptomatology. In the United States we are far from seeing or acknowledging any such affinity between the forms of fevers. This suggests the remark that, although it is becoming the fashion to disparage the annotating of English works by American editors, we believe useful additions might have been made on remittent fever, which is so imperfectly noticed under its diagnostic aspects by the author of the present volume, and also on yellow fever, which is not noticed at all. We must attribute to his imperfect view of the pathology of periodical fevers his separating remittent from intermittent fever, and placing them under different divisions, with eruptive fevers between them. His remarks on the diagnosis of both remittent and intermittent fevers leave unnoticed very important points in the diagnosis of those forms of periodical fever, so alarming in their appearance and often fatal in result, known in this country by the appellation of congestive, and on the continent of Europe by that of pernicious or malignant intermittent and remittent fevers.

Of fevers it is correctly remarked by the author, that "the period of incubation, as it is called, has no distinct character;" but he adds, "During the incursion of the fever, before any cutaneous eruption has appeared, there are certain indications which, more or less definitely, point to what is about to occur. 1. In measles it is attended with coryza. 2. In scarlatina there is sore throat, and the appearance of the tongue itself is peculiar. 3. In varioloid eruptions pain in the back is frequent. 4. Erysipelas is not marked by any special prodromata; there is a general sense of malaise, sometimes sore throat, and not unfrequently a dull, aching pain, or pricking sensation in the part that is to be attacked." Without undervaluing these indications, there are others overlooked in his enumeration which our own experience—and it has been tolerably large in all the forms of eruptive fevers—has strongly impressed on our memory. We cannot, for example, on the first appearance of the fever tell what face it is to assume, but we can tell with accuracy, in the larger number of cases that we have to deal with an eruptive fever, when we find our patient, especially if a child, laboring under a very hot skin, a pulse excessively frequent and wanting distinctness of beats, great restlessness and delirious agitation attended with somnolency, and for the most part some affection of the throat,—great dryness and rawness, or pain felt in deglutition. The agitation, frequency of pulse, and delirious wanderings, are not by any means in proportion to the fulness or sinister nature of the subsequent eruption. Thus, we have seen all these symptoms, combined with angina, in a greater degree of intensity in some mild cases of varioloid, or modified smallpox, than in what proved to be malignant and fatal ones of variola or primary unmitigated smallpox.

A demurrer will be entered by a large body of both writing and practising physicians against the introduction of epidemic cholera and influenza by the author into the category of febrile diseases, which latter he speaks of as constituting "a large class into which local disorder, as manifested by symptoms in particular organs, is only secondary and subsidiary to the general disease." In the instance of epidemic cholera, the local symptoms are as persistent and fully marked as they are in pneumonia and in cerebritis and hepatitis, while the fever is much less in cholera than in the phlegmasiæ just specified. In cholera there is not fever enough in the worst cases to allow of its being ranked with febrile diseases; nor are the organic lesions of such a kind as to give rise even to symptomatic fever, as in the phlegmasiæ. The author's chief reasons for the location he has assigned to cholera, are, "on account of the increasing conviction that it is one of the acute blood diseases, and the evident febrile reaction after recovery from the stage of collapse."

The volume before us contains a valuable chapter (V.) on rheumatism and gout, in reference to the diagnosis of these diseases, both separately and comparatively with each other. In relation to that of one division of the first mentioned, we read:—"There is little practical difficulty in recognizing a case of acute rheumatism; we have only to distinguish it from gout and from the inflammation of the joints attending on secondary deposit, and their diagnosis must be more fully considered in subsequent sections." Allusion is made to the researches of modern times which "have gradually led to the discovery of an important element in gout—the presence of an excess of uric acid." The author might as well, in this connection, have spoken of the kindred nature of gout and lithiasis, or the morbid secretory function of the kidney by which this organ gives issue to large quantities of lithic or uric acid.

We do not stop to criticise the classification of diseases adopted by Dr. Barclay, and which he has followed out in arranging his chapters on diagnosis. Classifications or any kind of systematic divisions can never, even in botany or zoology, be perfectly natural; but they must be received as helps to the memory, and as furnishing so many stand-points for farther, and, it may be, more extended observation. We shall, therefore, waive comments on the philosophy of placing chapter VI., on diseases of adventitious origin, after the one on gout and rheumatism, and preceding the chapters on dropsies and hemorrhages. The title of the sixth chapter conveys to the reader a very inadequate idea of its contents. These are thrown into two divisions, the first of which includes poisoning, vegetable, animal, and gaseous, together with syphilis, hydrophobia, glanders, and colica pictonum. We may remark, by the way, that neither in cause, symptoms, or treatment, can any affinity be observed between glanders and colica pictonum. The second division is made up of entozoa, echinococcus, and intestinal worms.

"Chronic blood ailments" constitute the materials, diagnostically considered, of chapter VIII. They are, under the first division, purpura and scurvy, anæmia, chlorosis, cachæmia or cachexia, pyæmia, and secondary deposits. The remarks on purpura and scurvy are very brief. Our knowledge of the first of these diseases has been enlarged by carefully made and recorded observations by German writers, who call it *blutkrankheit*, and the French, *hemophilie*, who give as its scientific cognomen, *hæmorrhagophilia*, or *hæmophilia*. (See *Archives Générales de Médecine*, Aug. 1857.) Touching cachæmia or cachexia, it is remarked, that there is one form which is well defined and of grave import: it is characterized by contamination of the blood with an admixture of pus; pyæmia or pyohæmia. Its probable source in all cases is the lining membrane of the veins, and it is constantly met with under circumstances in which there is no channel by which the pus globules could find their way into the circulation of the system. This disease is marked by fever of an adynamic type, quick, feeble pulse, dry, brown tongue, shivering often intense, followed by copious perspiration. "Cases of pyæmia bear a close analogy in many respects to glanders, and when the primary suppuration cannot be discovered they are somewhat perplexing. A sallow aspect and a peculiar odor of the breath have been both urged as characteristic of the disease; but while they may aid the diagnosis, they cannot be made the principal grounds of discrimination." Connected with this subject is thrombosis and embolia, or blood coagula and obstructions in the heart and blood-vessels, of which separate notice will be taken in another part of this journal.

Depraved Constitutional States is the heading of chapter IX., which has two divisions. 1. Scrofula and tuberculosis, including tabes mesenterica, phthisis, acute and chronic. Tubercles in the peritoneum and tubercles in the brain. 2. Morbid growths of different kinds and on different parts of the body.

The diagnosis of "quasi nervous diseases" is given in chapter X., in the sub-heading of which it is said, in reference to hysteria, that the evidence is almost entirely negative. "As the more frequent forms of hysteria are mere simulation of severe disease, so a regular hysterical paroxysm is, after its fashion, a simulation of epilepsy." Chorea, tetanus and delirium tremens are the other topics considered in this chapter.

Chapter XI. consists, in a few pages, of "a general examination of regions and organs. Then follow twenty-four chapters on the diagnosis of diseases of the brain, spinal cord, paralysis, neuralgia; examination of the chest, modifications of normal breath and voice sounds, and of percussion resonance, superadded sounds in their relation to altered breath and voice sounds; diseases of the respiratory organs; examination of the heart, diseases of the heart, diseases of the blood-vessels; diseases of the mouth and pharynx; examination of the abdomen; diseases of the intestinal canal; of the peritoneum; of the liver, spleen, and pancreas; examination of the urine; diseases of the urinary organs; of the ovaries; of the uterus; of the bones, joints, and muscles; of the skin and cellular tissue.

Restricted as we are to certain limits we can hardly do more than give this brief synopsis, which will serve, however, to convey to the reader an idea of the scope and the number and variety of diagnostic details of Dr. Barclay's volume. It was much wanted, and is full of instruction on a branch of pathology which furnishes we will not say the only, but certainly the chief and the safest, indications for the treatment of disease. Minute criticism might point out errors and omissions; but why should we dwell on these, when there is so much that is valuable and apposite to the wants of the student and practitioner? Were room allowed us for a comprehensive view of the subject, we might express a wish to see, in addition, a description of the aids to diagnosis and prognosis derived from an observation and study of the countenance of the patient, and the expression conveyed by it, as well as by each feature separately considered. One of the many pictures furnished in this way is often reproduced by writers, in describing the closing scene of life, under the title of the hippocratic countenance. From the eyes alone how much may be gathered for the indications of disease! It was well said by Hippocrates, the eyes tell the body's health:—"*Ita valet corpus sicuti valent oculi.*" These organs have been called the mirror of the soul; they are not less the mirror of health. So also of the position and attitudes in disease. They are full of meaning to the searching observer, and furnish material for pronouncing judgment in anticipation of analysis, of which indeed they are not always susceptible. An old nurse who has never gone beyond empirical routine may sometimes utter dicta in reference to the general expression and attitude of the sick which ought not to be unheeded by the young practitioner. Dr. Barclay has glanced at the subject: he will probably on a future occasion give it body and form.

There is yet another topic under the head of diagnosis which requires revision. We allude to the precise meaning or scientific value of the terms symptoms and signs, and of the attempts which have been made to separate them, so that symptoms or symptomatology shall be considered as furnishing the materials for diagnosis, and signs or semeiology or semeiotics, those for prognosis. The former, tell us the disease under which the patient is laboring; the latter, its probable course and its termination in health or in death—(Double—*Semeilogie Générale, ou Traité des Signes et de leur Valeur dans les Maladies,*)—while they would regard symptoms in the light just described, as rising and increasing with the lesion and only disappearing with this latter; and signs as the consequences deduced from the symptoms. A symptom may be spoken of as a good or a bad sign; but we cannot invert the proposition and say of a sign that it is a bad symptom. With Fernel we can say,—"*Omne symptoma signum est, non tamen omne signum symptoma.*" Celsus uses the word signs (*signa*) in connection with and as furnishing elements for prognosis. Baglivi employs the word with the same signification. We cannot speak of the return of appetite as a symptom of disease; but we can with entire accuracy hail it as a good sign. Involuntary discharges of fæces and urine is a symptom of a loss of innervation and of muscular debility; but in low fevers it is an alarming if not a fatal sign.

In conclusion: when looking at the symptoms of cerebral dyspepsia and the morbid distaste for sound medical literature and fundamental principles, accompanied with a relish for every empirical scrap and condiment, so prevalent throughout the land, we shall certainly hail, as a good sign, the favorable reception and careful study of Dr. Barclay's work on Medical Diagnosis.

